# Effects of melatonin on the nitric oxide system and protein nitration in the hypobaric hypoxic rat hippocampus

**DOI:** 10.1186/s12868-015-0199-6

**Published:** 2015-10-06

**Authors:** Chih-Chia Huang, Chia-Jou Lai, Mang-Hung Tsai, Ya-Chieh Wu, Kuang-Ti Chen, Ming-Jia Jou, Pin-I Fu, Ching-Hsiang Wu, I-Hua Wei

**Affiliations:** Department of Psychiatry, China Medical University Hospital, No. 91 Hsueh-Shih Road, Taichung, Taiwan; Institute of Clinical Medical Science, China Medical University, No. 91 Hsueh-Shih Road, Taichung, Taiwan; Department of Psychiatry, China Medical University, No. 91 Hsueh-Shih Road, Taichung, Taiwan; Institute of Basic Medical Science, China Medical University, No. 91 Hsueh-Shih Road, Taichung, Taiwan; Department of Anatomy, China Medical University, No. 91 Hsueh-Shih Road, Taichung, Taiwan; Department of Nursing, Ching-Kuo Institute of Management and Health, 336, Fu-Hsin Road, Keelung, Taiwan; School of Chinese Medicine for Post Baccalaureate, I Shou University, No. 1, Sec. 1, Syuecheng Road, Dashu District, Kaohsiung, Taiwan; Department of Anatomy and Cell Biology, College of Medicine, Taipei Medical University, 250 Wuxing Street, Taipei, Taiwan

**Keywords:** Hippocampus, Hypobaric hypoxia, Nitric oxide, Protein nitration, Melatonin

## Abstract

**Background:**

It is well documented that the nitric oxide (NO) might be directly involved in brain response to hypobaric hypoxia, and could contribute to memory deficiencies. Recent studies have shown that melatonin could attenuate hypoxia or ischemia-induced nerve injuries by decreasing the production of free radicals. The present study, using immunohistochemical and immunoblot methods, aimed to explore whether melatonin treatment may affect the expression of nitric oxide system and protein nitration, and provide neuroprotection in the rat hippocampus injured by hypobaric hypoxia. Prior to hypoxic treatment, adult rats were pretreated with melatonin (100 mg/kg, i.p.) before they were exposed to the altitude chamber with 48 Torr of the partial oxygen concentration (pO_2_) for 7 h to mimic the ambience of being at 9000 m in height. They were then sacrificed after 0 h, 1, and 3 days of reoxygenation.

**Results:**

The results obtained from the immunohistochemical and immunoblotting analyses showed that the expressions of neuronal nitric oxide synthase (nNOS), endothelial nitric oxide synthase (eNOS), inducible nitric oxide synthase (iNOS), nitrotyrosine (Ntyr) and Caspase 3 in the hypoxic hippocampus were increased from 0 h to 3 days of reoxygenation. Interestingly, the hypoxia-induced increase of nNOS, eNOS, iNOS, Ntyr and Caspase 3 protein expression was significantly depressed in the hypoxic rats treated with melatonin.

**Conclusions:**

Activation of the nitric oxide system and protein nitration constitutes a hippocampal response to hypobaric hypoxia and administration of melatonin could provide new therapeutic avenues to prevent and/or treat the symptoms produced by hypobaric hypoxia.

## Background

Sudden exposure to high altitude (HA) (i.e., rapid ascent without acclimatization, as in mountain climbing) results in the development of hypobaric hypoxia (HBH). Hypobaric hypoxia leads to appearance of neuropsychological disorders and mental dysfunctions such as insomnia, dizziness, and memory deficiencies which are consequences of the decreased partial pressure of oxygen (pO_2_) available to the central nervous system (CNS) [1, 2]. It has been established that CNS is highly sensitive to hypoxia and that some areas, such as the hippocampus, are especially vulnerable to hypoxic damage [3]. Several studies have determined that hypobaric hypoxia in the CA1 region of the hippocampus provokes metabolic, electrophysiological, and morphological modifications related to neuronal death [4–7]. The obvious cell damage in the hippocampus and learning/memory deficits were evidenced after exposure to HBH [8]. The exact mechanisms of neuronal damage in hypobaric hypoxia remain to be elucidated. Growing evidence showed that nitric oxide (NO) system involved in certain neuronal modifications and could contribute to memory deficiencies related to ischemic hypoxia, normobaric and hypobaric hypoxia [9–11]. NO is a short-lived bioactive molecule that participates in the physiology and pathophysiology of various systems in mammals. NO is produced by nitric oxide synthases (NOS) that constitute a family of enzymes that catalyze the oxidation of l-arginine and nicotinamide adenine dinucleotide phosphate by oxygen to yield L-citrulline and NO [12, 13]. Three distinct NOS isoforms have been identified: neuronal NOS (nNOS), endothelial NOS (eNOS), and inducible NOS (iNOS). It has been suggested that the formation of NO is directly linked to the glutamate, which can cause post-synaptic calcium influx and trigger a cascade of events leading to cell damage following *N*-methyl-d-aspartate (NMDA) receptors activation during hypoxia insults [14–16]. Part of the released NO after hypoxic injury can rapidly react with superoxide produced in excess during reoxygenation, forming peroxynitrite, a potent oxidizing agent with neurotoxic actions [17, 18]. Peroxynitrite can act on tyrosine residues in proteins to form the stable end product 3-nitro-l-tyrosine (nitrotyrosine; Ntyr). This compound can thus be used as a marker for the potentially cytotoxic effect of NO production in the presence of superoxide [18, 19].

Pharmacological agents can reduce NO production or prevent its biological effects by a variety of mechanisms, including the inhibition of l-arginine uptake into the cell, the reduction of cellular availability of necessary cofactors by preventing their formation or promoting their breakdown, or inhibition of the cellular mechanisms leading to induction of different NOS isoforms [12]. Of the many substances indentified, recent studies suggest that the melatonin and its metabolites are highly effective physiological antioxidants and free radical scavengers [20, 21]. Many biochemical and histopathological findings have revealed that melatonin exerts neuroprotective effects in suppressing NO production and enhancing superoxide dismutase (SOD) activity following numerous experimental and clinical oxidative injury [22–24]. Lowering circulating levels of melatonin also exaggerates the oxidative damage to tissues that are subjected to increased oxidative stress [25]. Thus, melatonin serving as a powerful agent in the treatment of various neurotoxicities is anticipated. To our knowledge, the potential effects of melatonin on nitric oxide system and protein nitration following hypobaric hypoxic insults in the hippocampus have not yet been explored. Therefore, this study was using immunohistochemical and immunoblot methods, aimed to explore the time course alteration of NOS and nitrotyrosine expression in the hippocampus of HBH rats. We also sought to elucidate whether melatonin treatment would have any beneficial effects to prevent and/or treat the symptoms associated with hypobaric disease.

## Methods

### Experimental animals

Healthy adult male Wistar rats weighing 150–250 g obtained from the Laboratory Animal Center of the National Taiwan University were used in this study. These animals were housed in conditions with controlled temperature (22 °C) and exposed to an automatically regulated light/dark cycle of 12:12 h (light on at 07:00–19:00 h), with ad libitum access to food and water throughout the study period. For the care and handling of all experimental animals, the guidelines as stated in the Guide for the Care and Use of Laboratory Animals (1985) as stated in the United States NIH guidelines (NIH publication No. 86-23), were followed. All the experiments were approved by our Laboratory Animal Center, China Medical University, Taiwan (No. 96-181-B). All efforts were made to minimize animal suffering and the smallest numbers of animal were used for the experiments presented. The experimental animals were carried out to evaluate the post-treatment effect of melatonin in hypoxic exposure. The animals (*n* = 180) were divided into four groups (I–IV) with 45 rats each. Groups I and II served as controls, the rats were subjected to normoxic breathing by receiving intraperitoneal administrations of vehicle (normal saline) and melatonin (100 mg/kg body weight in saline), respectively. Rats of groups III and IV were pretreated with intraperitoneal injections of normal saline and melatonin, respectively, 30 min before the hypobaric hypoxic insult. Melatonin (Sigma, St Louis, MO, USA) was dissolved freshly in pure absolute ethanol and later liquidized with isotonic sodium chloride (0.9 % NaCl) amounting to final concentration of 1:10 in a freshly prepared solution form, under sterile conditions. Pretreatment of both melatonin and normal saline was carried out at 10 am. Hypoxia was achieved by keeping the rats in an altitude chamber at 9000 m with the partial pressure of oxygen set at the level of 0.303 atm (pO_2_ = 48 Torr) for 7 h. Following hypobaric hypoxic exposure, each of the experimental groups was further divided into three subgroups (*n* = 15 each) sacrificed at 0 h, 1, and 3 days, respectively.

### Perfusion and tissue preparation

At each of the respective time points, both the hypoxic treated and control rats were deeply anesthetized with an intramuscular injection of mixtures of zoletil (30 mg/kg) and xylazine (10 mg/kg) and perfused transcardially with 100 ml of normal saline followed by 300 ml of 4 % paraformaldehyde in 0.1 M phosphate buffer (PB), pH 7.4. After perfusion, the hippocampus were removed and postfixed in the same fixative for 2 h. Tissue samples were then rinsed in 0.1 M PB and placed overnight in sucrose buffer (10–30 %) for cryoprotection at 4 °C. Serial 30 μm thick sections of the hippocampus were cut transversely with a cryostat (Bright 5040, Bright Instrument Company, Huntingdon, UK) on the following day and were alternatively placed into six wells of a cell culture plate, such that each well ultimately contained a group of sections, with each spaced a distance of 180 μm apart from the others. Sections collected in the first, second, and third wells were processed for nNOS, eNOS, and Ntyr immunohistochemistry.

### Immunohistochemistry

Following fixation and incubations as described above, sections collected in the wells were rinsed in 0.05 M Tris-buffer saline (TBS, pH 7.4), and then were treated in TBS containing 10 % methanol and 3 % hydrogen peroxide for 1 h to abolish the endogenous peroxidase activity. For blocking nonspecific binding, sections were first rinsed three times in TBS and then were reacted in an incubation medium containing 10 % normal goat serum or horse serum and 0.1 % Triton X-100 (all from Sigma) for 1 h. After several washes in TBS, the sections were then incubated separately in the primary monoclonal antibodies: nNOS (1:100; Santa cruz), Ntyr (1:3000; Santa cruz), and polyclonal antibodies: eNOS (1:1000; Santa cruz) overnight at 4 °C, respectively. After that they were treated separately with biotinylated horse anti-mouse and goat anti-rabbit antibodies (1:200; Vector) for 1 h at room temperature. After incubation in secondary antibody, the sections were processed by the standard Strepatavidin/HRP (DAKO) procedure with diaminobenzidine as a substrate of peroxidase.

### Western blot analysis

At a designated time point following experimental protocols rats from groups I–IV were deeply anesthetized and then the hippocampus were rapidly removed and kept in liquid nitrogen. After that, they were rinse with PBS and then were homogenized with 100 ml lysis buffer using a grinder on ice. For the tissue processing and western blot analysis, we followed the methods as described previously [26]. Briefly, 100 mg of solubilized proteins were separated by electrophoresis in a 10 % polyacrylamide gel, transferred to nitrocellulose membranes, and they were stained with Ponceau Red to confirm equal protein loading. The membranes were blocked with 5 % nonfat dry milk for 1 h, then immunoreacted with mouse monoclonal antibodies: nNOS (1:10,000; Santa cruz), Ntyr (1:500; Santa cruz), iNOS (1:1000; Santa cruz) and rabbit polyclonal antibodies: eNOS (1:1000; Santa cruz), Caspase 3 (1:1000; Millipore) overnight at 4 °C, respectively. The nitrocellulose sheet was further processed for chemiluminescence detection (Santa Cruz) using horseradish peroxidase (HRP)-conjugated anti-mouse, anti-rabbit and anti-sheep secondary antibodies (Santa Cruz) for 1 h at room temperature. Equal protein loading was confirmed by stripping the membranes, then immunoreacted with Beta-actin (1:1000; Sigma). Optical densities were quantified with a computer-assisted program (Gel-Pro Analyzer software).

### Nitrite assay

NO production was measured by the accumulation of nitrites (NO_2_^−^) in supernatant from different brain regions. The total amount of NO in hippocampus was assessed by the Griess reagent: 0.1 % *N*-(1-naphthyl) ethylene diamine dihydrochloride (Acros Organics), 1 % sulfanilamide (Cica) and 2.5 % H_3_PO_4_ (Cica) that detects nitrite, a stable reaction product of NO. Homogenates as described above for Western blotting were prepared and centrifuged at 15,000×*g* for 15 min and the supernatant was collected. The reagent was added to an equal volume of tissue supernatant (50 μl) and incubated for 10 min at room temperature. The optical density of each group was measured at 550 nm. Sodium nitrite dissolved in the lysis buffer was used as the standard.

### Quantitative study and image analysis

The nNOS, eNOS, and Ntyr staining was assessed in sections collected from the wells, and was quantified with a computer-based image analysis system (MGDS) along with the Image-Pro Plus software (Media Cybernetics, Silver Spring, MD, USA). A digital camera mounted on the Zeiss microscope imaged sections at 100× magnifications in bright field and displayed them on a higher resolution monitor. At this magnification the optical density (OD), which was used as an index to indicate labeling intensity, of reaction product in the cytoplasm of positive neurons, was measured by using a mouse to draw a line encircling the labeled soma on the digitized image. The OD of the background of each section was measured by averaging five random polygons (area of polygon = 150 μm^2^) with equal area of the neuropil of the hippocampus. The mean OD is the pixels that comprise the soma reading by densitometer. The actual amounts of staining intensity in a tissue section reflex the enzyme activity which is under the influence of multi-factors. Thus, all the parameters used in the present study were followed by Smolen’s method to ensure to gain a consistent result for gray level adjustment, histogram stretch and minimal optical density [27]. To avoid introducing bias two observers were blinded to examine the immunohistochemical sections for the image analysis for hippocampus. The OD of positive neurons at various time points in hypoxic animals, with or without melatonin pretreatment was subjected to a two-way ANOVA test. The data collected between the normoxic versus hypoxic groups at each time point were individually further analyzed using Student’s *t* test. Statistical difference was considered significant if *P* < 0.05.

### Control experiments

Some negative controls have been made to ensure the accuracy of nNOS, eNOS and Ntyr immunohistochemical results obtained from the present study. Thus, omission of nNOS, eNOS and Ntyr immunohistochemistry the primary and secondary antibodies in incubated reaction medium was carried out.

## Results

### Neuronal NOS immunoreactivity

**Fig. 1:**
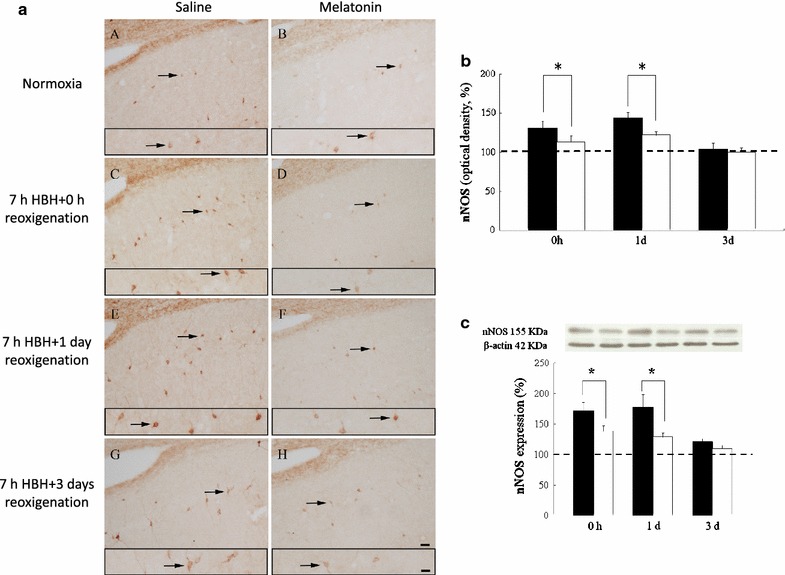
Hippocampal nNOS immunoreactivity in normoxic and hypobaric hypoxic rats pretreated with normal saline or melatonin, and sacrificed at 0 h, 1 and 3 days of reoxygenation. Light photomicrographs (**a**) show that light-stained nNOS immunoreactivity (*arrows*) is mainly found in pyramidal cells scattered throughout the CA1 region in normoxic rats (*A*, *B*). nNOS immunoreactivities are drastically increased at 0 h, 1 and 3 days after hypoxic exposure (*C*, *E*, *G*). In rats receiving melatonin pretreatment and subjected to 0 h- (*D*), 1- (*F*) and 3- (*H*) days hypoxic exposure, hippocampal nNOS immunoreactivity is significantly reduced. The *inserts* indicate nNOS(+) neurons of higher magnified in each representative figure. *Scale bar* 50 μm for all figures, *insert* 100 μm. *Histograms* showing the mean optical density of nNOS(+) neurons (**b**) and expression of total nNOS protein (**c**) quantified by immunoblots in the hippocampus of rats treated with hypoxia alone (*black column*) and melatonin pretreated (*white column*) and sacrificed at 0 h, 1 and 3 days of reoxygenation. Note that in hypoxic rats, the staining intensity and the levels of total protein of nNOS are drastically increased. In rats received melatonin, the nNOS staining and protein levels are successfully decreased in the hippocampus at the beginning and 1 day of reoxygenation. *Dashed line* shows the baseline controls are set as 100 % (saline or melatonin treatment under normoxic condition). The levels of β-actin are as a loading control (**c**). *P < 0.05 (Student’s t test) when compared with values (expressed as mean ± SEM) of rats treated only with hypoxia at the same survival time point

In normoxic rats that received normal saline or melatonin administration and sacrificed at various time points, the pattern of nNOS positive neurons detected in the hippocampal CA1 region was consistent; those nNOS positive neurons were weakly stained and predominantly distributed in the pyramidal cell layer of CA1 (Fig. 1a). At the same region of rats subjected to 7 h of HBH, an significantly increased of packing density and immunoreactive intensity of nNOS positive neurons was noticed (Fig. 1a). The majority of the nNOS positive neurons were heavily stained and the staining intensity was drastically enhanced to reach the peak level of 144 % after 1 day of reoxygenation following HBH (Fig. 1b). In rats with longer survival after hypoxic insult, nNOS immunoreactivity was decreased progressively. In the group of animals treated with the melatonin, the pattern and intensity of nNOS immunoreactivity were markedly reduced when compared with that of non-treated rats subjected to HBH. The increased immunoreactivity of nNOS positive neurons induced with HBH significantly was downregulated at 0 h and 1 day of reoxygenation and the prominent downregulation was sustained until 3 days of reoxygenation when the animals were treated with melatonin (Fig. 1b).

Western blot analysis of the hippocampus also revealed a marked increase of nNOS that reached the peak level of 178 % after 1 day of reoxygenation following HBH; these levels were declined to 121 % for those animals having longer survival times (at 3 days of reoxygenation following HBH) (Fig. 1c). In rats receiving melatonin pretreatment, the total nNOS protein levels were drastically decreased in rats surveyed at various time points when compared with those of the rats subjected to HBH but without melatonin pretreatment (P < 0.05; Fig. 1c). Results of nNOS immunoblots confirmed those of nNOS immunohistochemistry examination.

### Endothelial NOS immunoreactivity

**Fig. 2:**
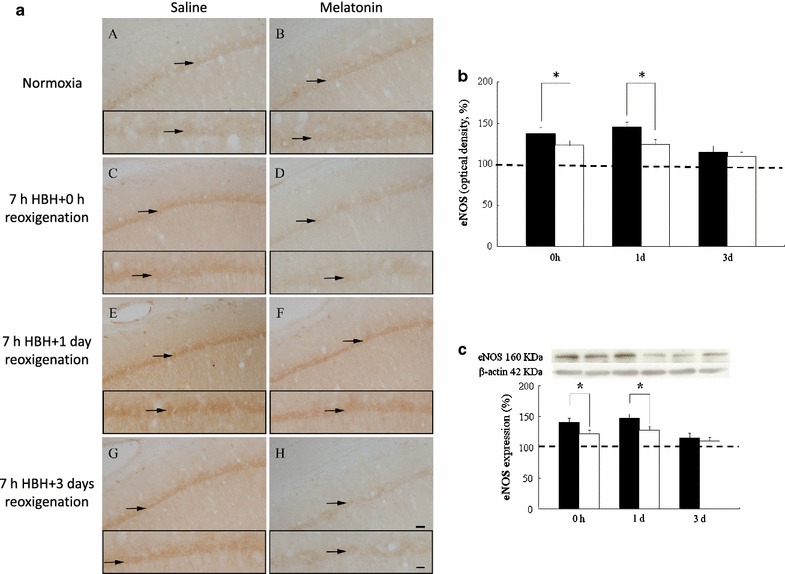
Hippocampal eNOS immunoreactivity in normoxic and hypobaric hypoxic rats pretreated with normal saline or melatonin and sacrificed at 0 h, 1 and 3 days of reoxygenation. In** a**, light photomicrographs show almost all pyramidal cells in the CA1 region exhibit weak eNOS immunoreactivity in the hippocampus of normoxic rats (*A*, *B*, *arrows*), the latter is drastically increased at 0 h (*C*), 1 (*E*) and 3 (*G*) days after hypoxic exposure. The augment of eNOS immunoreactivity at 0 h, 1 and 3 days post exposure is significantly declined in rats receiving melatonin pretreatment (*D*, *F*, *H*). eNOS(+) neurons of higher magnified in each representative figure are shown in the *inserts*. *Scale bar* 50 μm for all figures, *insert* 100 μm. Quantitative analyses showing the mean optical density of eNOS(+) neurons (**b**) and the level of total eNOS protein (**c**) quantified by immunoblots in the hippocampus of rats treated with hypoxia alone (*black column*) and melatonin pretreated (*white column*) and sacrificed at 0 h, 1 and 3 days of reoxygenation. The staining intensity and the levels of total protein of eNOS in the hippocamus are drastically enhanced in the rats sacrificed at 0 h, 1 and 3 days after hypoxic insult. In rats treated with hypoxia and pretreated melatonin, the increased intensity of eNOS stain and protein levels are markedly reduced as compared with those of hypoxic along. The staining intensity (**b**) or protein levels (**c**) of saline or melatonin treatment under normoxic condition rats are designed as controls (set as 100 %, indicated by *dashed line*). The levels of β-actin are as a loading control. *P < 0.05 (Student’s t test) when compared with values (expressed as mean ± SEM) of rats merely treated with hypoxia at the same survival time point

In the hippocampus from normoxic rats, eNOS immunoreactivity was observed occasionally in the pyramidal cells of CA1 and blood vessels that were lightly stained (Fig. 2a). After 7 h of HBH, an markedly augmented of cell density and immunoreactive intensity of eNOS positive neurons was noticed in the structures abovementioned. The staining intensity was drastically enhanced to reach the peak level of 145 % after 1 day of reoxygenation following HBH. After 3 days of reoxygenation following HBH, the distribution and intensity of eNOS immunoreactivity was decreased progressively (Fig. 2b). In rats receiving melatonin pretreatment prior to hypoxic insult, eNOS expression was markedly reduced when compared with that of rats with saline pretreatment. Melatonin pretreatment significantly downregulated HBH-induced increase in the immunoreactive intensity of eNOS positive neurons at 1 day of reoxygenation and the downregulation was sustained until 3 days of reoxygenation (Fig. 2b).

Western blot analysis of eNOS confirmed the results obtained from eNOS immunohistochemistry. Total eNOS protein levels in whole hippocampus were significantly increased to reach the peak level of 148 % after 1 day of reoxygenation following HBH and declined to 115 % for those animals at 3 days of reoxygenation following HBH (Fig. 2c). Melatonin pretreatment induced similar effect on the expression of eNOS protein that was drastically decreased in rats surveyed at various time points when compared with those of saline-pretreated rats subjected to HBH (P < 0.05; Fig. 2c).

### Inducible NOS immunoreactivity

**Fig. 3:**
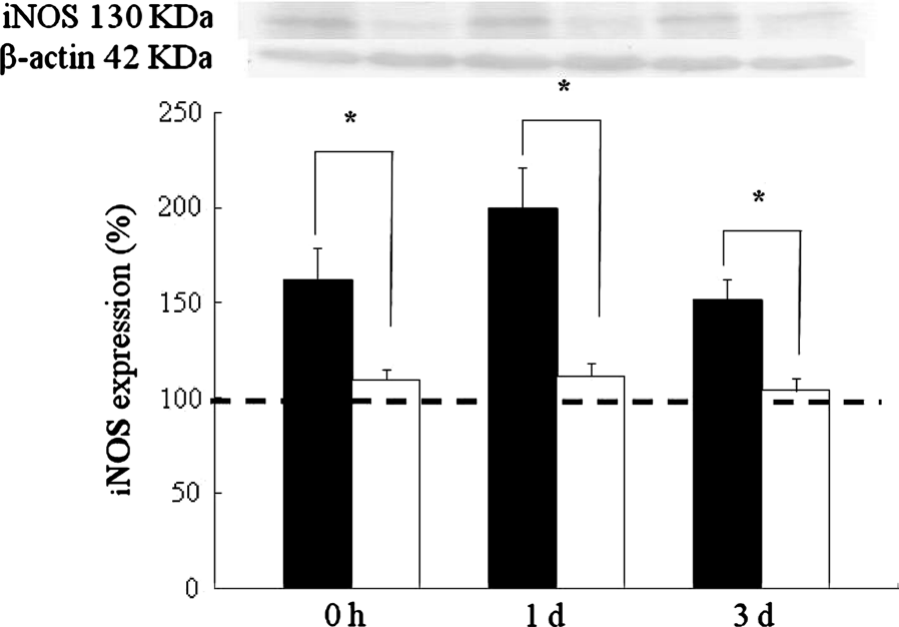
Expression of total iNOS proteins quantified by immunoblots in whole hippocampus homogenates of rats treated with hypoxia alone (*black column*) and melatonin pretreated (*white column*) and sacrificed at 0 h, 1 and 3 days of reoxygenation. The levels of total iNOS protein in the hippocamus are drastically increased in the rats sacrificed at 0 h, 1 and 3 days after hypoxic insult. In rats treated with hypoxia and pretreated melatonin, the increased protein levels are markedly reduced as compared with those of hypoxic along. The protein expression of saline or melatonin treatment under normoxic condition rats are designed as controls (set as 100 %, indicated by *dashed line*), which are normalize with the levels of β-actin as a loading control. *P < 0.05 (Student’s t test) when compared with values (expressed as mean ± SEM) of rats merely treated with hypoxia at the same survival time point

Total iNOS protein levels in whole hippocampus were significantly increased to reach the peak level of approximately 200 % after 1 day of reoxygenation following HBH and declined to 151 % for those animals at 3 days of reoxygenation following HBH (Fig. 3). Similar trend was evidenced in melatonin pretreatment on the expression of iNOS protein that was drastically decreased in rats surveyed at various time points when compared with those of saline-pretreated rats subjected to HBH (P < 0.05; Fig. 3).

### Nitrotyrosine immunoreactivity

**Fig. 4:**
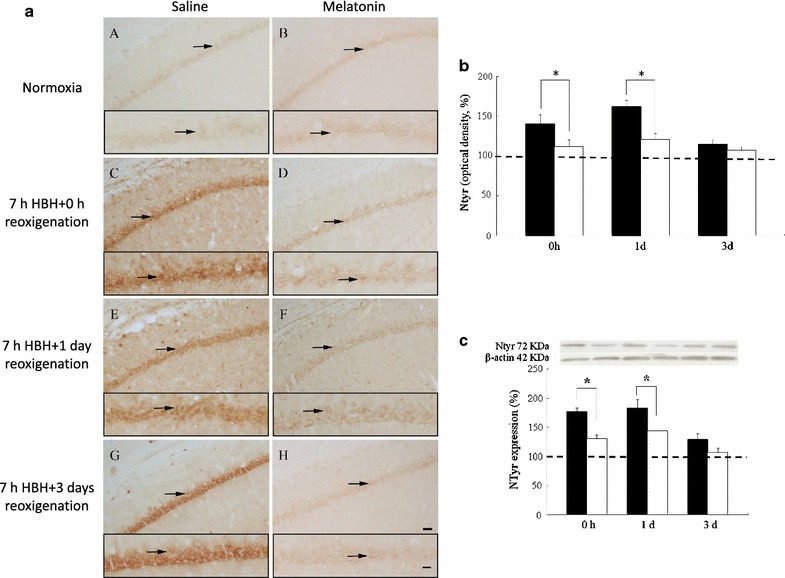
Hippocampal Ntyr immunoreactivity in normoxic and hypobaric hypoxic rats pretreated with normal saline or melatonin, and sacrificed at 0 h, 1 and 3 days of reoxygenation. Light photomicrographs (**a**) show that majority of pyramidal cells in the CA1 region of normoxic rats are weak Ntyr immunoreactive (*A*, *B*, *arrows*) but significantly increased in immunoreactivity following hypoxic exposure for 0 h (*C*), 1 (*E*) and 3 (*G*) days. Melatonin pretreatment appreciably decreases Ntyr immunoreactivity boosted at 0 h (*D*), 1 (*F*) and 3 (*H*) days after hypoxic exposure. Ntyr(+) neurons are magnified in each representative figure and shown in the *inserts*. *Scale bar* 50 μm for all figures, *insert* 100 μm. Quantitative results are shown the mean optical density of Ntyr(+) neurons (**b**) and the level of total Ntyr protein (**c**) quantified by immunoblots in the hippocampus of rats treated with hypoxia alone (*black column*) and melatonin pretreated (*white column*) and sacrificed at 0 h, 1 and 3 days of reoxygenation. The staining intensity and the levels of total protein of Ntyr in the hippocamus are drastically increased in the rats sacrificed at 0 h, 1 and 3 days after hypoxic insult. The enhanced intensity of Ntyr stain and protein levels are markedly reduced in rats treated with hypoxia and pretreated melatonin as compared with those of hypoxic along. Controls (set as 100 %, indicated by *dashed line*) are the staining intensity (**b**) or protein levels (**c**) of saline or melatonin treatment under normoxic condition rats. Loading control is the levels of β-actin. *P < 0.05 (Student’s t test) when compared with values (expressed as mean ± SEM) of rats merely treated with hypoxia at the same survival time point

The rats hippocampus contained Ntyr immunoreactive neurons that were few in number and distributed in the pyramidal layer of CA1. In the normoxic condition, Ntyr immunopostive pyramidal neurons contained less amount of the protein (Fig. 4a). In rats subjected to 7 h of HBH, pyramidal neurons drastically augmented in number and immunoreactivity. The latter reached the peak level of 162 % after 1 day of reoxygenation following HBH. In rats with longer survival after hypoxic insult, Ntyr immunoreactivity was declined gradually (Fig. 4b). After 3 days of reoxygenation following HBH, the distribution and intensity of the Ntyr immunoreactivity declined as found in normoxic condition. The pretreatment of melatonin again showed similar effect on the downregulation of HBH-induced Ntyr expression to those of nNOS and eNOS (Figs. 1b, 2b, 4b).

The findings of Ntyr immunohistochemistry were then confirmed by Western blot analysis that also showed a significant increase of Ntyr after early reoxygenation and a decline in those animals having longer survival times (Fig. 4c). The blot analysis of Ntyr also revealed a similar trend of the changes of Ntyr expression in rats receiving melatonin pretreatment and was parallel to those of nNOS, eNOS and iNOS (Figs. 1c, 2c, 3, 4c).

### Nitrite assay

**Fig. 5:**
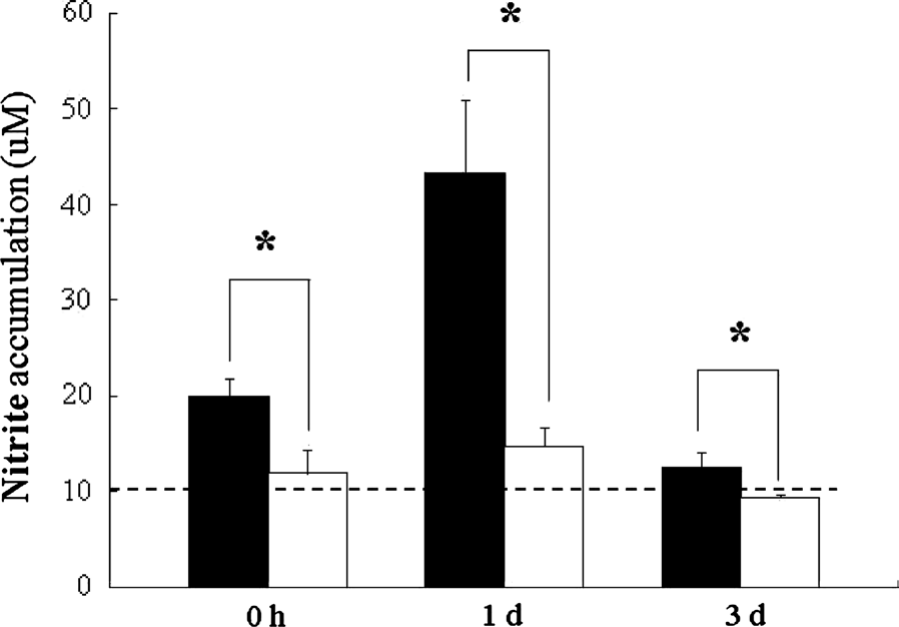
NO production levels in the hippocamus of rats treated with hypoxia alone (*black column*) and melatonin pretreated (*white column*) and sacrificed at 0 h, 1, and 3 days of reoxygenation. After hypoxic insult, the NO levels in rat hippocampus are significantly increased. In hypoxic rats pretreated with melatonin, the levels of NO production are markedly reduced as compared with those of rats treated only with hypoxia. The NO levels of saline or melatonin treatment under normoxic condition rats are designed as controls (100 %, indicated by *dashed line*). *P < 0.05 (Student’s t test) when compared with values (expressed as mean ± SEM) of rats simply treated with hypoxia at the same survival time point

In normoxic rats that received normal saline or melatonin administration, the whole hippocampus possessed few amount of NO (Fig. 5). In rats subjected to 7 h of HBH, a significant increase of NO production was noticed, reached the peak level of 43 μM after 1 day of reoxygenation following HBH and decreased gradually in rats with longer survival after hypoxic insult (Fig. 5). Downregulation of HBH-induced NO production was significantly observed in animals pre-treated with melatonin and persistent until 3 days of reoxygenation (P < 0.05; Fig. 5).

### Caspase 3 immunoreactivity

**Fig. 6:**
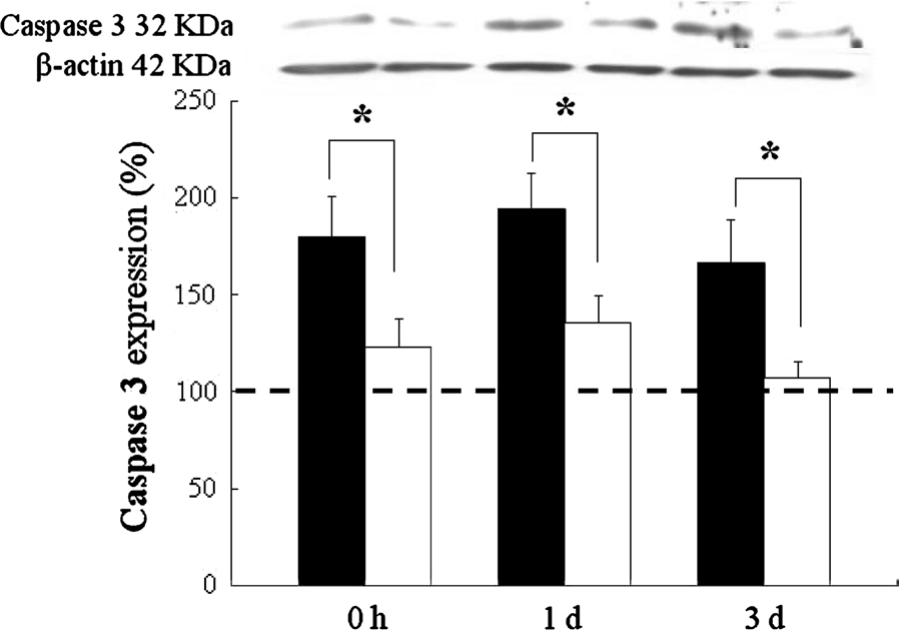
Expression of total Caspase 3 proteins quantified by immunoblots in whole hippocampus homogenates of rats treated with hypoxia alone (*black column*) and melatonin pretreated (*white column*) and sacrificed at 0 h, 1 and 3 days of reoxygenation. Note that at 0 h, 1 and 3 days after hypoxic insult, the rats show drastically increased levels of total Caspase 3 protein in the hippocamus. In those rats treated with hypoxia and pretreated melatonin, the increased protein levels are markedly reduced as compared with those of hypoxic along. The protein expression of saline or melatonin treatment under normoxic condition rats are designed as controls (set as 100 %, indicated by *dashed line*), which are normalize with the levels of β-actin as a loading control. *P < 0.05 (Student’s t test) when compared with values (expressed as mean ± SEM) of rats merely treated with hypoxia at the same survival time point

As Caspase 3 is known to play a central role in the execution-phase of cell apoptosis pathway, the levels of Caspase 3 were measured. Our results showed that HBH increased the levels of Caspase 3 in rat hippocampus following HBH (Fig. 6). The HBH-induced elevation of Caspase 3 levels were also reduced by pretreatment of melatonin (P < 0.05; Fig. 6).

## Discussion

The current study provides the information concerning the melatonin may attenuate HBH induced expression of the nitric oxide system and protein nitration in the hippocampus. We selected the hippocampus as a target for our study considering that the hippocampal susceptibility to hypoxic damage is well established [7, 28]. However, the relationship between exposure to HBH and modifications of the nitric oxide system following melatonin treatment in the hippocampus has rarely been studied, although some reports suggest that NO may be involved in physiological and pathological responses after climbing and air travel as well as in migration to higher altitudes [11]. We report here that HBH boosts NO production and upregulates nNOS, eNOS and iNOS. There is also a modulation of Ntyr and Caspase 3 immunoreactivity that parallels to nNOS, eNOS and iNOS expression after HBH. In addition, our previous and other studies have shown that some antioxidants such as melatonin, green tea and hypoxic strategies may play roles to enhance endogenous antioxidative defense systems to prevent biological organisms from oxidative injuries [29–37]. Interestingly, melatonin selected as an antioxidant for this study showed that melatonin markedly dampened HBH-induced increases in the expressions of nNOS, eNOS and iNOS, NO production and Ntyr formation. Melatonin also effectively protects neurons against HBH-induced neuronal damage in the hippocampus. This protection is supported by a decrease of Caspase 3 levels. The present immunohistochemical results in control and experimental rats subjected to HBH were corroborated by Western blot analysis and NO production measurements.

It is known that in the hippocampus, NO is involved in several physiologic events as neural plasticity, acting as a retrograde messenger in the case of long-term potentiation and participating in the creation of memory [38–40]. In addition, NO is also involved in physiopathology, playing a role in neuronal damage. Previous evidences that supported a neurotoxic role after hypoxic-ischemic injury in the brain, cerebral cortex, cerebellum, hippocampus and nodose ganglion due to the over-expression of NOS [11, 31, 33–36, 41–43]. hypoxic-ischemic injury causes cell damage in the hippocampus has been associated with memory loss, functional and behavioral deficits [44, 45]. Constitutive NOS expression increased after hypoxic-ischemic damage [11, 33, 34, 42] was also detected in our present study. This increase probably constitutes part of the cascade occurring after hypoxic injury, including glutamate release, calcium influx, activation of NOS, NO synthesis, and reaction with resulted oxygen radicals [46–48]. The over-production of NO or peroxynitrite (ONOO^−^) could readily trigger a series of biochemical reactions to modulate enzyme activities and subsequently lead to lipid peroxidation or DNA damage [48, 49]. Thus the fact that NO is protective or destructive depends on its amount in the organisms; small amounts might have beneficial effects to protect against neuronal damage, and large amounts, which may produce by enhanced activation of NOS, might have detrimental effects to cause cell damage or cell death. The present results showed that HBH induced a significantly increased in nNOS, eNOS and iNOS immunoreactivity and in protein expression in the hippocampus following 0 h and 1 days of reoxygenation. This elevation decreased progressively after 3 days of reoxygenation. The increase in NO production, Ntyr formation and Caspase 3 level followed the same pattern. These results are in agreement with those of Encinas et al. (2003) who found an increase in NOS expression and NO production in the hippocampus immediately after acute hypoxia applied in a hypobaric chamber at 8325 m (260 mmHg) and our previous findings that chronic treadmill running protects hippocampal neurons from hypobaric hypoxia-induced neruonal injury in rats [50]. Although nNOS was reported to be mainly responsible for brain cNOS activity and play the neurotoxic role after hypoxic-ischemic injury in the brain, cerebral cortex, hippocampus and nodose ganglion [11, 31, 33, 34, 41, 42], however, our major finding showed that eNOS immunoreactivity detected in hippocampal pyramidal cells of CA1 was also augmented and peaked after 1 day of reoxygenation following 7 h of HBH. The eNOS expression in hippocampal pyramidal cells of CA1 is coherent with other studies [51, 52]. The eNOS-derived NO has been suggested as a retrograde messenger of long-term potentiation and implicated in synaptic plasticity [51]. It may also be involved in mitochondrial dysfunction and subsequent pathological changes of prion diseases [52]. Based on the present findings, it is therefore likely that upregulation of eNOS contributes to the incremented cNOS activity and provides a neurotoxic role for NO. Furthermore, the Western blot analysis showed the HBH induced iNOS protein expression in the hippocampus at 0 h, 1 and 3 days post hypoxia. Treatment with melatonin resulted in down-rgulation of the iNOS expression and attenuation of the surge of NO in hippocampus due to HBH. The NO burst as a result of iNOS upregulation during acute hypobaric hypoxia interrupts the memory consolidation had been reported [53]. The present study provided further evidences on the sources of NO surge that are multiply derived from nNOS, eNOS and iNOS.

Another consequence of NO production is the generation of nitrotyrosine. NO reacts with superoxide radicals producing peroxynitrite, a new and powerful oxidant with the capacity of nitrating tyrosine residues, thus forming nitrotyrosine, a direct marker of NO synthesis and peroxynitrite formation [48]. In the present model of HBH, coincident with the peaks of NO production and the expressions of nNOS and eNOS is an increase in nitrotyrosine formation. Overproduction of oxygen free radicals including superoxide has been shown in neurons after hypoxia and ischemia [54, 55], which in addition to the overproduction of NO, results in a situation to produce peroxynitrite of cytotoxic concentration. Peroxynitrite has been shown to nitrate tyrosine residues in proteins [18], causing consequent protein-structure changes and alterations of enzymatic activities [19, 56]. Furthermore, mitochondrial proteins have been shown to be a selective target for peroxynitrite, an event directly related to neuronal damage [18]. Our previous studies have shown that inappropriate or excessive NOS expression and NO production are coincident with the death of affected neurons following hypoxia and peripheral nerve injury [33–36]. In this connection, the amount of NOS reactivity and NO levels may be set as an index for the severity of neuronal damage. By reducing the parameters of NOS activity, NO production and Caspase 3 levels could decrease neurological signs and neuronal damage. This detrimental role can be inhibited by the application of melatonin in the nervous tissue. The antioxidative properties of melatonin and its metabolites have been extensively studied and the use of this molecule as a novel neural protector has been widely reported [25, 31, 57–61]. In the present study, coherent with our previous studies [30–32], we further demonstrated the effective high dosage of melatonin (100 mg/kg) to achieve protection against HBH-induced increase in NO production, expressions of nNOS, eNOS and iNOS, and Ntyr formation in the hippocampus. The neuroprotective functions of melatonin are directly attributed to its antioxidant properties and free radical scavenging ability [59, 62]. Intracellular melatonin can bind to calmodulin, which conceivably would suppress the calmodulin-dependent nitric oxide synthase activity [63]. Besides reducing NO formation by restricting the activation of NOS and thereby limiting the subsequent cytotoxicity caused by this free radical, melatonin was recently shown to directly scavenge the highly toxic NO and ONOO^−^ anion as well [64]. Thus, with the reduction in NO synthesis, melatonin can also protect neurons by its ability to scavenge NO as well as ONOO^−^ and associated oxidants.

## Conclusion

In summary, this study has demonstrated that hypoxia-induced increases of NO production, nNOS, eNOS and iNOS expressions as well Ntyr formation and Caspase 3 levels in the hippocampus is effectively prevented by melatonin treatment. This novel finding has not only helped to achieve a better understanding of the functional roles of NO involved in the processes of neuronal damage, but also offered the possibilities of potential therapeutic use of melatonin for the preventing and/or treating the symptoms associated with hypobaric exposure.

## Abbreviations

HA: high altitude
HBH: hypobaric hypoxia
pO_2_: partial oxygen concentration
CNS: central nervous system
NO: nitric oxide
NOS: nitric oxide synthases
nNOS: neuronal nitric oxide synthase
eNOS: endothelial nitric oxide synthase
iNOS: inducible nitric oxide synthase
NMDA: *N*-methyl-d-aspartate
Ntyr: 3-nitro-l-tyrosine (nitrotyrosine)
SOD: superoxide dismutase
OD: optical density
ONOO^−^: peroxynitrite

## References

[1] Bahrke MS, Shukitt-Hale B (1993). Effects of altitude on mood, behaviour and cognitive functioning. A review. Sports Med (Auckland, NZ).

[2] Maiti P, Singh SB, Sharma AK, Muthuraju S, Banerjee PK, Ilavazhagan G (2006). Hypobaric hypoxia induces oxidative stress in rat brain. Neurochem Int.

[3] Pulsinelli WA (1985). Selective neuronal vulnerability: morphological and molecular characteristics. Prog Brain Res.

[4] Trouvin JH, Prioux-Guyonneau M, Cohen Y, Jacquot C (1986). Rat brain monoamine metabolism and hypobaric hypoxia: a new approach. Gen Pharmacol.

[5] Krapivin SV, Romanova VE, Voronina TA, Luk’ianova LD (1991). An electrophysiological study of the brain of rats with different resistances to oxygen deficiency in acute hypoxia. Fiziologicheskii zhurnal SSSR imeni I M Sechenova.

[6] Shukitt-Hale B, Kadar T, Marlowe BE, Stillman MJ, Galli RL, Levy A, Devine JA, Lieberman HR (1996). Morphological alterations in the hippocampus following hypobaric hypoxia. Hum Exp Toxicol.

[7] Maiti P, Muthuraju S, Ilavazhagan G, Singh SB (2008). Hypobaric hypoxia induces dendritic plasticity in cortical and hippocampal pyramidal neurons in rat brain. Behav Brain Res.

[8] Titus AD, Shankaranarayana Rao BS, Harsha HN, Ramkumar K, Srikumar BN, Singh SB, Chattarji S, Raju TR (2007). Hypobaric hypoxia-induced dendritic atrophy of hippocampal neurons is associated with cognitive impairment in adult rats. Neuroscience.

[9] Beckman JS (1991). The double-edged role of nitric oxide in brain function and superoxide-mediated injury. J Dev Physiol.

[10] Matsuoka Y, Kitamura Y, Tooyama I, Kimura H, Taniguchi T (1997). In vivo hypoxia-induced neuronal damage with an enhancement of neuronal nitric oxide synthase immunoreactivity in hippocampus. Exp Neurol.

[11] Encinas JM, Serrano J, Bentura ML, Castro-Blanco S, Fernandez AP, Rodrigo J (2003). Nitric oxide system and protein nitration are modified by an acute hypobaric hypoxia in the adult rat hippocampus. J Neuropathol Exp Neurol.

[12] Moncada S, Palmer RM, Higgs EA (1991). Nitric oxide: physiology, pathophysiology, and pharmacology. Pharmacol Rev.

[13] Snyder SH, Bredt DS (1992). Biological roles of nitric oxide. Sci Am..

[14] Goldberg IH (1987). Free radical mechanisms in neocarzinostatin-induced DNA damage. Free Radic Biol Med.

[15] Bredt DS, Snyder SH (1992). Nitric oxide, a novel neuronal messenger. Neuron.

[16] Brenman JE, Bredt DS (1997). Synaptic signaling by nitric oxide. Curr Opin Neurobiol.

[17] Koppenol WH, Moreno JJ, Pryor WA, Ischiropoulos H, Beckman JS (1992). Peroxynitrite, a cloaked oxidant formed by nitric oxide and superoxide. Chem Res Toxicol.

[18] Beckman JS, Koppenol WH (1996). Nitric oxide, superoxide, and peroxynitrite: the good, the bad, and ugly. Am J Physiol.

[19] Ischiropoulos H, Al-Mehdi AB (1995). Peroxynitrite-mediated oxidative protein modifications. FEBS Lett.

[20] Siu AW, Maldonado M, Sanchez-Hidalgo M, Tan DX, Reiter RJ (2006). Protective effects of melatonin in experimental free radical-related ocular diseases. J Pineal Res.

[21] Tan DX, Manchester LC, Terron MP, Flores LJ, Reiter RJ (2007). One molecule, many derivatives: a never-ending interaction of melatonin with reactive oxygen and nitrogen species?. J Pineal Res.

[22] Montilla PL, Tunez IF, Munoz de Agueda C, Gascon FL, Soria JV (1998). Protective role of melatonin and retinol palmitate in oxidative stress and hyperlipidemic nephropathy induced by adriamycin in rats. J Pineal Res.

[23] Okatani Y, Wakatsuki A, Kaneda C (2000). Melatonin increases activities of glutathione peroxidase and superoxide dismutase in fetal rat brain. J Pineal Res.

[24] Reiter RJ, Tan DX, Terron MP, Flores LJ, Czarnocki Z (2007). Melatonin and its metabolites: new findings regarding their production and their radical scavenging actions. Acta Biochim Pol.

[25] Reiter RJ, Tan DX, Cabrera J, D’Arpa D (1999). Melatonin and tryptophan derivatives as free radical scavengers and antioxidants. Adv Exp Med Biol.

[26] Encinas JM, Fernandez AP, Salas E, Castro-Blanco S, Munoz P, Rodrigo J, Serrano J (2004). Nitric oxide synthase and NADPH-diaphorase after acute hypobaric hypoxia in the rat caudate putamen. Exp Neurol.

[27] Smolen AJ, Conn PME (1990). Image analysis techniques for quantification of immunohistochemical staining in the nervous system. Quantitative and qualitative microscopy methods in neuroscience.

[28] Shukitt-Hale B, Stillman MJ, Welch DI, Levy A, Devine JA, Lieberman HR (1994). Hypobaric hypoxia impairs spatial memory in an elevation-dependent fashion. Behav Neural Biol.

[29] Simon DK, Standaert DG (1999). Neuroprotective therapies. Med Clin N Am..

[30] Chang HM, Ling EA, Lue JH, Wen CY, Shieh JY (2000). Melatonin attenuates neuronal NADPH-d/NOS expression in the hypoglossal nucleus of adult rats following peripheral nerve injury. Brain Res.

[31] Chang HM, Ling EA, Chen CF, Lue H, Wen CY, Shieh JY (2002). Melatonin attenuates the neuronal NADPH-d/NOS expression in the nodose ganglion of acute hypoxic rats. J Pineal Res.

[32] Chang HM, Huang YL, Lan CT, Wu UI, Hu ME, Youn SC (2008). Melatonin preserves superoxide dismutase activity in hypoglossal motoneurons of adult rats following peripheral nerve injury. J Pineal Res.

[33] Wei IH, Wu YC, Wen CY, Shieh JY (2004). Green tea polyphenol (−)-epigallocatechin gallate attenuates the neuronal NADPH-d/nNOS expression in the nodose ganglion of acute hypoxic rats. Brain Res.

[34] Wei IH, Huang CC, Chang HM, Tseng CY, Tu HC, Wen CY, Shieh JY (2005). Neuronal NADPH-d/NOS expression in the nodose ganglion of severe hypoxic rats with or without mild hypoxic preconditioning. J Chem Neuroanat.

[35] Wei IH, Huang CC, Tseng CY, Chang HM, Tu HC, Tsai MH, Wen CY, Shieh JY (2008). Mild hypoxic preconditioning attenuates injury-induced NADPH-d/nNOS expression in brainstem motor neurons of adult rats. J Chem Neuroanat.

[36] Wei IH, Tu HC, Huang CC, Tsai MH, Tseng CY, Shieh JY (2011). (−)-Epigallocatechin gallate attenuates NADPH-d/nNOS expression in motor neurons of rats following peripheral nerve injury. BMC Neurosci.

[37] Wu UI, Mai FD, Sheu JN, Chen LY, Liu YT, Huang HC, Chang HM (2011). Melatonin inhibits microglial activation, reduces pro-inflammatory cytokine levels, and rescues hippocampal neurons of adult rats with acute Klebsiella pneumoniae meningitis. J Pineal Res.

[38] Bohme GA, Bon C, Stutzmann JM, Doble A, Blanchard JC (1991). Possible involvement of nitric oxide in long-term potentiation. Eur J Pharmacol.

[39] Bohme GA, Bon C, Lemaire M, Reibaud M, Piot O, Stutzmann JM, Doble A, Blanchard JC (1993). Altered synaptic plasticity and memory formation in nitric oxide synthase inhibitor-treated rats. Proc Natl Acad Sci USA.

[40] Estall LB, Grant SJ, Cicala GA (1993). Inhibition of nitric oxide (NO) production selectively impairs learning and memory in the rat. Pharmacol Biochem Behav.

[41] Eliasson MJ, Huang Z, Ferrante RJ, Sasamata M, Molliver ME, Snyder SH, Moskowitz MA (1999). Neuronal nitric oxide synthase activation and peroxynitrite formation in ischemic stroke linked to neural damage. J Neurosci Off J Soc Neurosci.

[42] Serrano J, Encinas JM, Fernandez AP, Rodrigo J, Martinez A (2006). Effects of acute hypobaric hypoxia on the nitric oxide system of the rat cerebral cortex: protective role of nitric oxide inhibitors. Neuroscience.

[43] Serrano J, Encinas JM, Salas E, Fernandez AP, Castro-Blanco S, Fernandez-Vizarra P, Bentura ML, Rodrigo J (2003). Hypobaric hypoxia modifies constitutive nitric oxide synthase activity and protein nitration in the rat cerebellum. Brain Res.

[44] Sinden JD, Rashid-Doubell F, Kershaw TR, Nelson A, Chadwick A, Jat PS, Noble MD, Hodges H, Gray JA (1997). Recovery of spatial learning by grafts of a conditionally immortalized hippocampal neuroepithelial cell line into the ischaemia-lesioned hippocampus. Neuroscience.

[45] Hartman RE, Lee JM, Zipfel GJ, Wozniak DF (2005). Characterizing learning deficits and hippocampal neuron loss following transient global cerebral ischemia in rats. Brain Res.

[46] Cazevieille C, Muller A, Meynier F, Bonne C (1993). Superoxide and nitric oxide cooperation in hypoxia/reoxygenation-induced neuron injury. Free Radic Biol Med.

[47] Love S (1999). Oxidative stress in brain ischemia. Brain Pathol (Zurich, Switzerland)..

[48] Szabo C (1996). The pathophysiological role of peroxynitrite in shock, inflammation, and ischemia-reperfusion injury. Shock (Augusta, Ga).

[49] Moncada C, Arvin B, Le Peillet E, Meldrum BS (1991). Non-NMDA antagonists protect against kainate more than AMPA toxicity in the rat hippocampus. Neurosci Lett.

[50] Lin C, Wu CJ, Wei IH, Tsai MH, Chang NW, Yang TT, Kuo YM (2013). Chronic treadmill running protects hippocampal neurons from hypobaric hypoxia-induced apoptosis in rats. Neuroscience.

[51] Dinerman JL, Dawson TM, Schell MJ, Snowman A, Snyder SH (1994). Endothelial nitric oxide synthase localized to hippocampal pyramidal cells: implications for synaptic plasticity. Proc Natl Acad Sci USA.

[52] Park JH, Kim BH, Park SJ, Jin JK, Jeon YC, Wen GY, Shin HY, Carp RI, Kim YS (2011). Association of endothelial nitric oxide synthase and mitochondrial dysfunction in the hippocampus of scrapie-infected mice. Hippocampus.

[53] Udayabanu M, Kumaran D, Nair RU, Srinivas P, Bhagat N, Aneja R, Katyal A (2008). Nitric oxide associated with iNOS expression inhibits acetylcholinesterase activity and induces memory impairment during acute hypobaric hypoxia. Brain Res.

[54] Daval JL, Ghersi-Egea JF, Oillet J, Koziel V (1995). A simple method for evaluation of superoxide radical production in neural cells under various culture conditions: application to hypoxia. J Cereb Blood Flow Metabol Off J Int Soc Cereb Blood Flow Metabol.

[55] Forman LJ, Liu P, Nagele RG, Yin K, Wong PY (1998). Augmentation of nitric oxide, superoxide, and peroxynitrite production during cerebral ischemia and reperfusion in the rat. Neurochem Res.

[56] Eiserich JP, Estevez AG, Bamberg TV, Ye YZ, Chumley PH, Beckman JS, Freeman BA (1999). Microtubule dysfunction by posttranslational nitrotyrosination of alpha-tubulin: a nitric oxide-dependent mechanism of cellular injury. Proc Natl Acad Sci USA.

[57] Dziegiel P, Murawska-Cialowicz E, Jethon Z, Januszewska L, Podhorska-Okolow M, Surowiak P, Zawadzki M, Rabczynski J, Zabel M (2003). Melatonin stimulates the activity of protective antioxidative enzymes in myocardial cells of rats in the course of doxorubicin intoxication. J Pineal Res.

[58] Manda K, Anzai K, Kumari S, Bhatia AL (2007). Melatonin attenuates radiation-induced learning deficit and brain oxidative stress in mice. Acta Neurobiol Exp.

[59] Reiter RJ, Acuna-Castroviejo D, Tan DX, Burkhardt S (2001). Free radical-mediated molecular damage. Mechanisms for the protective actions of melatonin in the central nervous system. Ann NY Acad Sci.

[60] Reiter RJ, Tan DX, Qi W, Manchester LC, Karbownik M, Calvo JR (2000). Pharmacology and physiology of melatonin in the reduction of oxidative stress in vivo. Biol Signals Recept.

[61] Vural H, Sabuncu T, Arslan SO, Aksoy N (2001). Melatonin inhibits lipid peroxidation and stimulates the antioxidant status of diabetic rats. J Pineal Res.

[62] Tomas-Zapico C, Caballero B, Sierra V, Vega-Naredo I, Alvarez-Garcia O, Tolivia D, Rodriguez-Colunga MJ, Coto-Montes A (2005). Survival mechanisms in a physiological oxidative stress model. FASEB J Off Publ Fed Am Soc Exp Biol.

[63] Pozo D, Reiter RJ, Calvo JR, Guerrero JM (1997). Inhibition of cerebellar nitric oxide synthase and cyclic GMP production by melatonin via complex formation with calmodulin. J Cell Biochem.

[64] Blanchard B, Pompon D, Ducrocq C (2000). Nitrosation of melatonin by nitric oxide and peroxynitrite. J Pineal Res.

